# Extravascular factor IX after gene therapy in hemophilia B, does it matter?

**DOI:** 10.1016/j.rpth.2025.102723

**Published:** 2025-03-06

**Authors:** Quentin Van Thillo, Cédric Hermans

**Affiliations:** 1 Haemophilia Centre, Department of Cardiovascular Sciences, University Hospitals Leuven, Leuven, Belgium; 2 Thrombosis and Haemostasis Unit, Division of Haematology, Cliniques universitaires Saint-Luc, Brussels, Belgium

**Keywords:** biological availability, cross-reactive material, extended FIX half life product, factor IX, Extravascular space, genetic therapy

## Abstract

Gene therapy will very likely change the treatment paradigm of hemophilia B in the coming years. For the majority of patients, adjunctive exogenous factor (F)IX clotting factor concentrate will continue to be needed in case of surgery or bleeding. However, there is insufficient evidence on the optimal FIX product to be used in these circumstances, given the differences in body distribution between the currently available products. Unknown factors include the behavior of FIX Padua in the extravascular space and its contribution to hemostasis. Other issues are the potential importance of the presence of cross-reactive material and the discrepancies between different assays in measuring FIX activity. In conclusion, even after gene therapy, the differences between different FIX products remain relevant for optimal bleeding and perioperative management. Thus, real-world data on the use of exogenous FIX after gene therapy are needed to determine the preferred exogenous FIX concentrate.

## Essentials

• Gene therapy does not fully protect most persons with hemophilia B in case of trauma or surgery.
• The optimal adjunctive factor IX product after gene therapy has not yet been determined.
• The role of cross-reactive material and the body distribution of the product should be evaluated.
• More real-world data are needed on the use of exogenous factor IX after gene therapy.

Gene therapy is transforming the treatment of hemophilia B. Etranacogene dezaparvovec (Hemgenix, CSL Behring) and fidanacogene elaparvovec (Durveqtix or Beqvez, Pfizer) have received conditional authorization from the European Medicines Agency and approval from the US Food and Drug Administration and are already commercially available in multiple countries. These gene products are based on adeno-associated viruses and make use of the more potent factor (F)IX Padua variant. The latest updated findings from the HOPE-B [1] and BENEGENE-2 [2] trials show a mean FIX level, as measured by the one-stage assay, of 38.6% (median, 36.0%; range, 4.8%-80.3%) at 3 years and 26.9% (median, 22.9%; range, 1.9%-119.0%) at 15 months, respectively, thereby approaching the nonhemophilia range. Most patients remain free from spontaneous bleeding and no longer require clotting FIX concentrate in prophylaxis. However, since 67% of patients in the HOPE-B trial had FIX levels below 40% after 2 years of follow-up [3], the majority of patients after gene therapy will likely need additional clotting factor concentrate in the advent of traumatic bleeding or around major invasive procedures. Currently, it is not known though which adjunctive FIX product would be preferred in these situations.

Several concentrates of FIX are available today, including plasma-derived FIX, recombinant standard half-life FIX nonacog alfa (Benefix, Pfizer), and recombinant extended half-life (EHL) FIX products. EHL products consist of recombinant FIX (rFIX), which is linked to a protein that confers EHL. FIX is linked to either the Fc fragment of immunoglobulin G1 eftrenonacog alfa or rFIX-Fc (Alprolix, Sobi), or albumin albutrepenonacog alfa or rFIX-FP (Idelvion, CSL Behring), leading to recycling via the neonatal Fc receptor back into the circulation, thereby delaying lysosomal degradation. A third EHL product consists of rFIX linked to polyethylene glycol nonacog beta pegol or N9-GP (Refixia, Novo Nordisk), delaying degradation through various physiological elimination processes. In prophylaxis, the EHL products allow good bleeding control with fewer intravenous injections (from once weekly to once every 3 weeks) and have become the standard of care [[4], [5], [6]].

However, several important considerations specific to FIX should be made. First, FIX is a much smaller molecule than FVIII. It is well established from various preclinical models that FIX exits in the circulation and then resides within the subendothelial basement membrane by binding to collagen IV. This way, there is a reservoir of FIX in case of injury to the endothelium, allowing fast reentry of FIX into the circulation and subsequent amplification of the coagulation cascade that is triggered by the extrinsic pathway. Both Benefix and Alprolix bind to collagen IV with similar affinity. In addition, FIX colocalizes with collagen I in the knee joints of mice [7], and Alprolix is able to bind to the neonatal Fc receptor in the subendothelial space [8]. Recently, it was shown in a mouse tail clip bleeding model that a variant of FIX (rFIX_5KR_) with increased affinity for collagen IV offered increased hemostatic protection [9], highlighting the importance of extravascular FIX and its binding to collagen in hemostasis.

Since FIX Padua does not influence the size of the protein, we can safely assume that after gene therapy, the newly produced FIX Padua is subject to the same body distribution as physiological, unmodified FIX, but this has not yet been studied. One may speculate that most of the FIX Padua steadily produced by transfected liver cells following gene therapy is present extravascularly as a stable but not measurable FIX pool. Accordingly, in gene therapy patients, the saturation level of the extravascular FIX binding sites should range from around 5% to normal, reflecting the patient’s intravascular FIX activity. This likely would contrast with the fluctuating intra- and extravascular concentrations of FIX resulting from repeated prophylactic intravenous administrations. This is also in contrast with hemophilia A, where the entire intravascular pool of FVIII produced following gene therapy is readily measurable in the blood compartment. Moreover, it is unknown whether the Padua variant affects the function of FIX in the extravascular space or displays an altered affinity to collagen IV. Nevertheless, a different binding affinity is highly unlikely because the mutation only concerns a single amino acid and is far removed from the collagen-binding domain (Figure).

**Figure:**
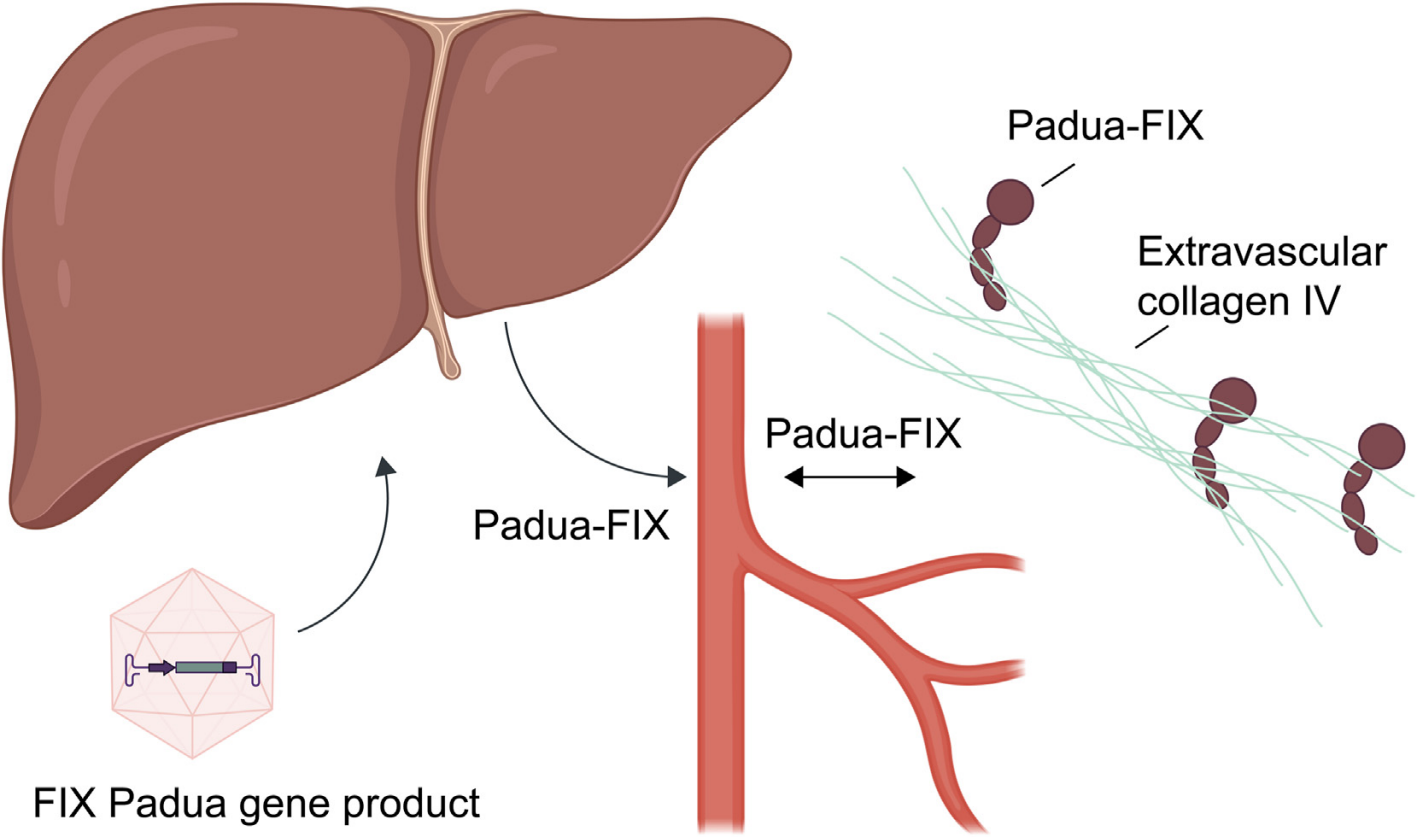
The extravascular pool of factor (F)IX plays an important role in hemostasis. Gene therapy consists of the hyperactive FIX Padua transgene, which is delivered to the hepatocytes using an adeno-associated viral vector. FIX Padua is subsequently produced by the liver and enters the circulation. Similar to endogenous FIX, FIX Padua then supposedly exits the circulation and binds to collagen IV in the extravascular space, thereby providing a stable extravascular reservoir of FIX Padua.

The EHL FIX product that, according to current understanding, best replicates this physiological role of FIX is eftrenonacog alfa because the fusion to Fc does not increase the size to the same extent as fusion to either albumin or polyethylene glycol. This explains the lower trough levels that are observed after administration of rFIX-Fc, but also the lack of correlation between trough levels and bleed rates in some patients on EHL products, with observations of break-through bleeds in some patients despite trough levels above 10% [10,11].

Second, in the majority of cases, hemophilia B is caused by missense mutations, whereas in many persons with hemophilia A, no FVIII protein is present because of inversions. This implies that dysfunctional residual fragments of FIX can be found in many persons with hemophilia B, which are called cross-reactive material (CRM). This CRM is able to compete with exogenously delivered FIX for binding to collagen IV in the subendothelial matrix. CRM-negative patients require less frequent prophylaxis [8]. There is currently no evidence of the effect of CRM status on the response to gene therapy. Whether different dosing of exogenous FIX should be used in patients after gene therapy, depending on their CRM status, remains to be assessed. It is likely that the newly produced FIX Padua will constantly and steadily compete with CRM for binding sites in the subendothelial matrix. However, it is unknown whether the extravascular space is saturated with FIX or whether additional extravascular FIX is needed for adequate hemostasis after gene therapy.

A third issue is the wide variation in FIX activity measurement using the one-stage assays with different activators and chromogenic assays with both over- and underestimation after infusion of EHL FIX products [12]. On top of this, there are some discrepancies between the one-stage assay and chromogenic assay in measuring FIX activity for FIX Padua. The FIX activity after gene therapy is measured by the one-stage assay in most studies and is considered superior for FIX Padua [13]. However, the optimal assay to assess FIX activity in patients receiving exogenous FIX after gene therapy has not yet been evaluated. Moreover, strategies that estimate the extravascular pool of FIX are currently not available. The best way to have an indirect idea about the distribution is through measuring the recovery of FIX. Therefore, we advise performing a comprehensive pharmacokinetic analysis the first time when exogenous FIX is administered after gene therapy.

Based on these considerations, we anticipate that EHL factor products with a smaller distribution volume and limited extravascular transfer might provide better hemostatic control in case of bleeding or perioperatively when higher plasma levels of FIX are more relevant. Extravascular FIX seems more important for the prevention of day-to-day bleeds, whereas intravascular FIX levels are important for controlling traumatic bleeds. Based on these assumptions, we would be inclined to recommend using rFIX-FP over rFIX-Fc after gene therapy to achieve hemostasis in case of surgery or trauma. Treatment efficacy should then be followed, measuring peak and trough levels to estimate the amount of intravascular FIX. However, whether these theoretical differences between FIX concentrate products are clinically relevant is highly uncertain and should be studied in trials. The results of invasive procedures, including 9 major surgeries, after etranacogene dezaparvovec were recently reported by O’Connell et al. [14]. These interventions could be managed with low amounts of exogenous FIX and were not associated with thrombotic adverse events. However, there was no information on CRM status or the body distribution of exogenous FIX [14]. At our centers, 2 patients who were included in the HOPE-B trial underwent major surgery.

The first patient, at St-Luc Hospital, suffered from hemophilia B with an underlying insertion c.199_200insAluY. Based on this insertion, which results in a frameshift in the γ-carboxyglutamate (Gla) domain [15], the patient is supposedly CRM-negative. At the age of 43 years, he was treated with etranacogene dezaparvovec in 2019. After gene infusion, FIX activity was around 20% at 4 weeks, with a drop to 15% at week 5 with accompanying alanine transaminase elevation, after which corticosteroids were given as per protocol. From month 6 to 24, FIX expression was maintained at 9.6% to 12.9%. By month 30, the FIX level had declined to 3.6% with no apparent causative etiology (normal liver tests and no inflammation). Prophylactic treatment was resumed on day 857 postdosing with rFIX-Fc (Alprolix) at a dose of 100 International Units (IU)/kg every 10 days. In September 2024, the patient underwent an abdominoplasty, for which he received a preoperative bolus of rFIX-Fc 100 IU/kg (9000 Units), achieving a FIX level of 75% perioperatively. Postoperative complications included significant hemorrhage with a drop in hemoglobin levels from 15 g/L to 8.7 g/L after 24 hours. After 48 hours, he underwent a revision surgery with a preoperative peak level of 98%. However, perioperative bleeding necessitated the transfusion of 2 units of red blood cells in total.

A second patient at the University Hospitals of Leuven underwent a knee arthroplasty in February 2024. He was supposedly CRM-positive based on a missense mutation (g.31344 T>C, p.Ile454Thr). The patient had a baseline FIX activity level of 52.3%, more than 4 years after infusion of etranacogene dezaparvovec. To minimize the bleeding risk, he received 24 IU/kg of rFIX-Fc (Alprolix, 2000 IU) prior to surgery and after 24 and 48 hours, on top of tranexamic acid. He had a good recovery with a peak level of 79.9%, which could be explained by either competition with the dysfunctional endogenous FIX in the extravascular space or saturation of the extravascular pool with FIX Padua (Table).

**Table:** Overview of the hemostatic management of 2 patients undergoing major surgery after etranocogene dezaparvovec.

Time (h)	Patient 1 (Saint-Luc)			Patient 2 (UZ Leuven)		
	FIX pre (%)	FIX post (%)	Dose (IU)	FIX pre (%)	FIX post (%)	Dose (IU)
0	7	75	9000	52.3	79.9	2000
24	40		4000	60.2		2000
48	52	98	8000	98.9		2000
72		64	5000	127.2		-
96		103	5000	-		-

The FIX levels at trough (pre) and peak (post) are shown. The dose of exogenous FIX is indicated, and both patients received recombinant FIX-Fc (Alprolix).

FIX, factor IX; IU, International Units; UZ, University Hospitals Leuven.

In summary, even after gene therapy, the differences between the different FIX products likely remain relevant for optimal bleeding and perioperative management. Therefore, we want to raise attention to specific issues associated with FIX supplementation after gene therapy in hemophilia B. To address these questions, we consider it essential to collect all data from postmarket authorization studies on the use of gene therapy. Data should be collected within trials evaluating the use of exogenous FIX after gene therapy, including CRM status, pharmacokinetic measurements, and clinical outcomes. Moreover, preclinical studies evaluating the impact of FIX Padua in the subendothelial matrix are needed to gain more insight into its extravascular role in hemostasis. This way, more light could be shed on the optimal use of FIX products after gene therapy.
